# Safety and Clinical Outcome of Bleomycin-Electrosclerotherapy (BEST) Treating Lymphatic Malformations (LMs)

**DOI:** 10.1007/s00270-025-04169-6

**Published:** 2025-09-04

**Authors:** Florian Obereisenbuchner, Elena Borisch, Daniel Puhr-Westerheide, Sinan Deniz, Ferdi Cay, Mirjam Schirren, Gloria Biechele, Robin Schregle, Beate Häberle, Julia Haehl, Alexandra Hartl, Alexandra Fröba-Pohl, Fatemeh Kashani, Jens Ricke, Max Seidensticker, Moritz Wildgruber, Vanessa F. Schmidt

**Affiliations:** 1 Department of Radiology, LMU University Hospital, LMU Munich, Marchioninistraße 15, 83177 Munich, Germany; 2 Interdisciplinary Center for Vascular Anomalies (IZGA), LMU University Hospital, LMU Munich, Munich, Germany; 3 Department of Radiology, Hacettepe University, Ankara, Turkey; 4 Institute of Micro Technology and Medical Device Technology, Technical University Munich, Munich, Germany; 5 Department of Paediatric Surgery, LMU University Hospital, LMU Munich, Munich, Germany; 6 Department of Otorhinolaryngology, LMU University Hospital, LMU Munich, Munich, Germany

**Keywords:** Bleomycin-electrosclerotherapy, BEST, Slow-flow vascular malformation, Lymphatic malformation, LM, Sclerotherapy

## Abstract

**Introduction:**

Bleomycin-electrosclerotherapy (BEST) is a novel treatment for slow-flow vascular malformations (SFVMs), most studied in venous malformations. This study specifically evaluated its safety and clinical outcome in lymphatic/lymphatic-dominant lympho-venous malformations (LMs/ld-LVMs).

**Materials and Methods:**

A monocentric cohort with symptomatic LMs or ld-LVMs treated by BEST was retrospectively assessed. A treatment-specific, patient-reported questionnaire assessed overall clinical response (complete response: symptom-free, partial response: improved symptoms, no response: unchanged symptoms, progression of symptoms), subjective health-related quality of life (QoL; optimal, improved, unchanged, worsening), pain (numerical rating scale, NRS), and postprocedural skin discolouration (yes/no). Pre and postprocedural lesion size was measured in three planes on MRI.

**Results:**

Twenty-seven treatments were performed in 20 patients with 14 LMs and six ld-LVMs (11 microcystic, five mixed, and four macrocystic subtypes). Patients received 1.4 ± 0.6 treatments with a median bleomycin dose of 7 mg (range 2–15 mg). After BEST, 7/20 (35%) patients reported complete response, 10/20 (50%) partial response, and 3/20 (15%) no response. Health-related QoL was stated as optimal in 10/20 (50%) and as improved in 4/20 (20%) patients. Median pain NRS was reduced from 6 (3–10) to 2 (0–6). Postprocedural skin discolouration occurred in 11/20 (55%) patients. Follow-up MR imaging revealed lesion size reduction from mean maximum volume of 793 cm^3^ (IQR 155–2199 cm^3^) to 548 cm^3^ (IQR 71–1059 cm^3^). Total complication rate (CIRSE grade 3–4) was 11.1%. No differences in all outcome parameters regarding LMs subtypes were assessed.

**Conclusion:**

BEST demonstrates efficacy and acceptable safety treating LMs and ld-LVMs, including challenging microcystic lesions previously considered difficult to treat.

**Level of Evidence:**

3b, Retrospective Cohort Study.

**Graphical Abstract:**

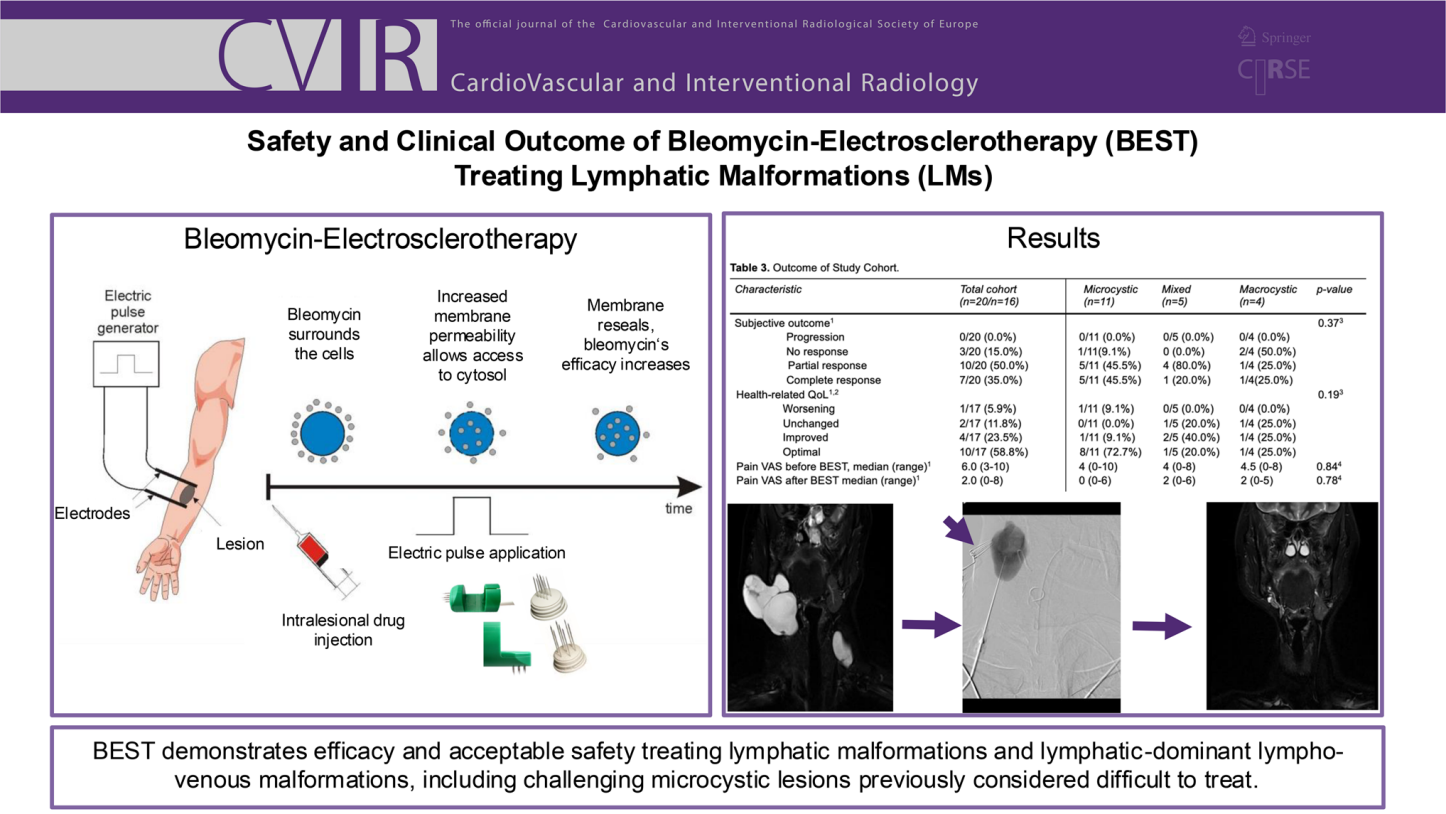

**Supplementary Information:**

The online version contains supplementary material available at 10.1007/s00270-025-04169-6.

## Introduction

Congenital vascular anomalies are among the most complex vascular disorders. Lymphatic malformations (LMs) are the second most common type of slow-flow vascular malformations [1]. They can rapidly enlarge and be blue coloured in case of intermittent infections or internal haemorrhage. Functional impairment of adjacent structures can lead to pain and swelling, while disfigurement of affected areas is frequently demonstrated [2, 3]. According to the 2025 International Society for the Study of Vascular Anomalies (ISSVA) classification [4], LMs are further divided into isolated LMs (micro, macrocystic, and mixed), complex LMs, and lymphedemas [4]. LMs with macrocystic components are frequently managed surgically, particularly when complete resection is feasible. Alternatively, percutaneous sclerotherapy, in which a sclerosant is injected directly into the lesion to induce endothelial inflammation, fibrosis, and ultimately obliteration of the cysts [1, 5]. Extensive LMs often require multiple sessions, while microcystic LMs respond less to sclerotherapy; therapeutic challenges thus remain [6–8]. Recently, Bleomycin-electrosclerotherapy (BEST) has emerged as a novel therapeutic approach for the treatment of SFVMs. This technique combines reversible electroporation, which temporarily increases cell membrane permeability, with intralesional administration of bleomycin, resulting in a several-fold increase in intracellular drug concentration [9–11]. First retrospective studies have demonstrated promising results for the treatment of SFVMs, and early evidence also supports its potential efficacy in LMs [12–17]. Within this study, we focus specifically on the application of BEST in LMs and assess both safety and patients’ outcomes. 

## Materials and Methods

### Study Design

Patients were recruited from the interdisciplinary vascular anomalies centre at a tertiary care hospital. Data were collected using electronic patient records and the picture archiving and communication system (PACS). Diagnosis of a LM or lymphatic-dominant lymphovenous malformation (ld-LVM) was based on patient history, physical examination, (duplex-)ultrasound (US), and a dedicated contrast-enhanced magnetic resonance imaging (MRI). The latter included fast spin echo (FSE) T1-weighted (T1w), short tau inversion recovery (STIR), and fast 3D gradient-echo (GRE) T1w sequences. On imaging, LMs and ld-LVMs have been defined as multiloculated cystic lesions with fluid-like signal, no internal flow voids, and typically enhancement only of cyst walls and septa. Macrocystic subtypes were determined as large, well-defined cysts (> 2 cm) with fluid-filled cavities on MRI. Microcystic subtypes have been identified as diffuse, composed of numerous small cysts (< 2 cm) that may appear solid-like and often involve skin or mucosa. Mixed subtypes were defined as a combination of both.

All LMs and ld-LVMs treated with BEST from 2022 until 2024 were included. Interdisciplinary vascular board decisions for BEST were made following discussions among at least one interventional radiologist, a paediatrician/paediatric surgeon in case of the patient being < 18 years old, one otolaryngologist/ENT surgeon for head-and-neck lesions as well as an angiologist. Indications for BEST were made based on the symptom complex (swelling, bleeding, recurring infections, pain, aesthetic disfigurement, and accompanying functional impairment).

Selection criteria for BEST were untreated lesions not considered suitable for conventional sclerotherapy (e.g., microcystic LMs) as well as therapy-refractory or recurrent LMs defined as lesions that persisted or worsened clinically and/or radiologically despite prior invasive treatment. Exclusion criteria for BEST were pregnancy, lactation, already reached lifetime dose of 100 mg of Bleomycin (or 1.3 mg/kg for children) in line with the updated current operating procedure (COP) for BEST [11]. In women of childbearing age, ß-HCG was tested, and patients were informed to avoid conception during 6 months after BEST. As the most feared complication of bleomycin is pulmonary toxicity, all patients were assessed clinically (included history of past pulmonary disease) for acute or chronic lung injury. In case of positive history or clinical finding, pulmonary function tests and chest X-ray were obtained.

### Procedural Details

For detailed description of the procedural details, see Supplemental 1.

### Follow-Up

Patients were regularly scheduled for a standardized follow-up regime. The first clinical follow-up was routinely performed at 3–6 months after each BEST session. BEST was repeated if patients presented with both an incomplete improvement of symptoms and residual cystic LM compartments being visible on follow-up imaging. Standard follow-up included patient history, clinical examination, and MRI. The clinical parameters assessed were changes in pain intensity and frequency, swelling, functional impairment, bleeding/ulceration, cosmetic disfigurement, and frequency of recurrent superinfections or erysipelas.

### Assessment of Patients’ Outcome

For evaluation of subjective outcomes, treatment-specific patient-reported questionnaires (see Supplemental 2) were utilized, completed 3–6 months after treatment including patients ’ rating of overall clinical response and health-related quality of life (QoL) as well as grading of pain pre and postprocedural using a numeric rating scale (NRS), and documentation of postprocedural skin discolourations. In detail, the overall clinical response was classified into four categories (complete response: symptom-free, partial response: improved symptoms, no response: unchanged symptoms, progression of symptoms). For the subjective rating of health-related QoL, four categories (optimal, improvement, unchanged, and worsening) were used. Postprocedural skin discolouration was recorded dichotomously (yes/no). The further course of occurred skin changes during follow-up was classified by the patient as unchanged, reduced, or fully resolved. Imaging outcome was evaluated by changes in lesion size on STIR MRI sequences before and after BEST. Therefore, lesion volume was calculated as the product of maximum diameters in the axial, coronal, and sagittal plane.

### Procedure-Related Complications

All reported peri or postinterventional complications were categorized according to CIRSE classification system [18]. Since skin discolouration following BEST is a known and frequent side effect [11, 16], patients (and parents) were consented in detail about this prior to treatment, while these manifestations were not classified as complications. Respiratory surveillance after BEST is routinely performed according to COP [11], and in case, a patient developed respiratory symptoms, post-treatment assessment of pulmonary function is performed. Special attention was given to the documentation of further skin alterations (e.g., necrosis and blisters) and peripheral nerve damage (e.g., paresis, sensory, and motor disturbances) as well as wound complications and bleeding.

### Statistical Analysis

Descriptive statistics as well as statistical testing were performed using R-Studio (version 2024.09.0 + 375) and R (version 4.3.2). Alpha was set to < 0.05. Descriptive statistics were used to analyse the distribution of patients among the different categories. The Shapiro test was used to assess the normality of data distribution. Data are presented as mean ± standard deviation, median (range), or median (interquartile range), as appropriate. Wilcoxon ranked sum test was used to compare imaging results and Chi-squared test to assess clinical response. For subgroup analysis of the different types of LMs, Kruskal–Wallis test and Wilcoxon signed-rank tests were used.

## Results

### Patient Characteristics

**Fig. 1 A:**
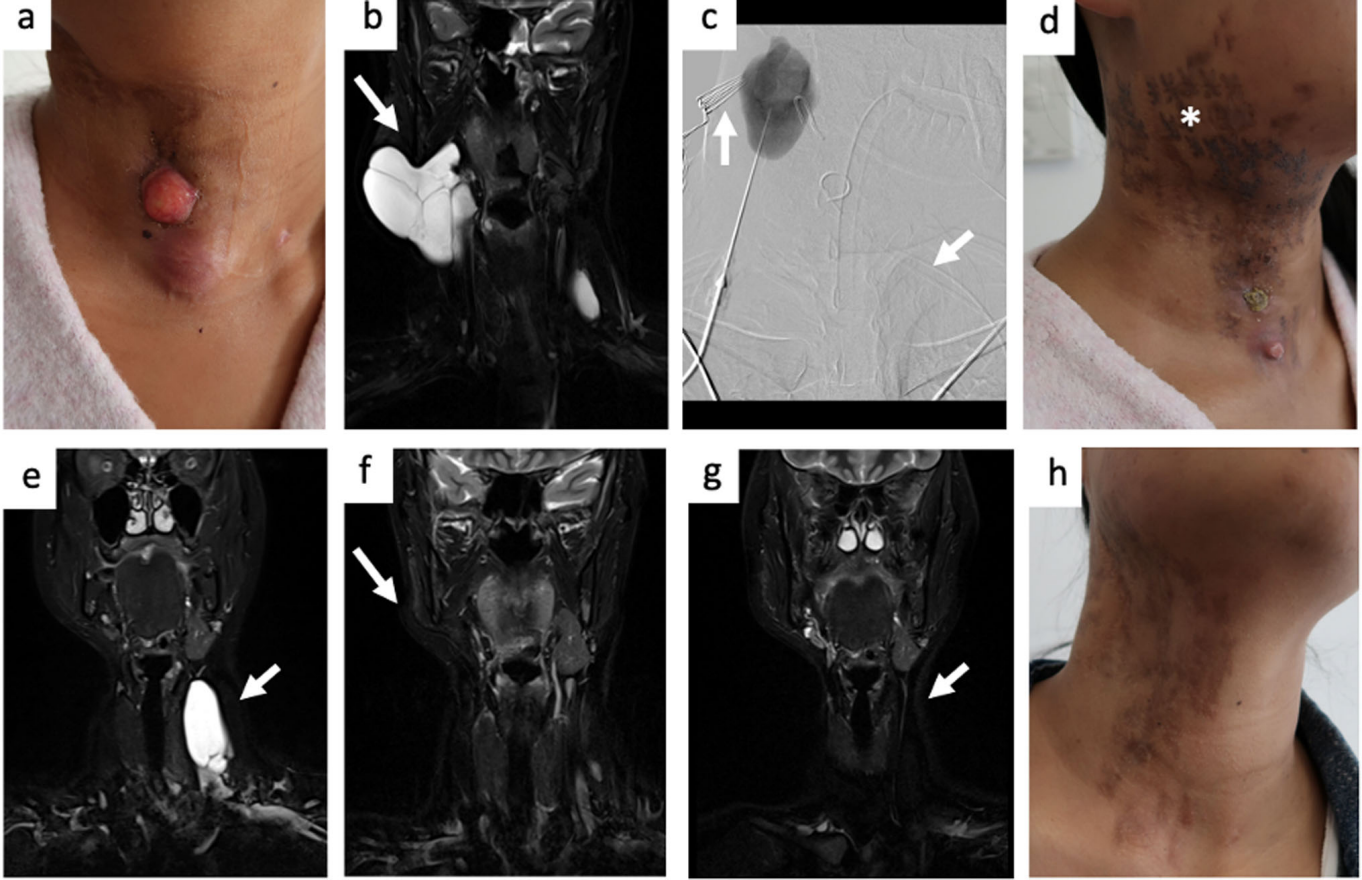
30-year-old female patient with macrocystic lymphatic malformations (LMs) of the neck treated by two sessions of Bleomycin-electrosclerotherapy (BEST) **a** Clinical photograph before BEST treatment; note the swelling as well as the superficial localized components spontaneously ruptured some weeks before. **b** Coronal short-tau-inversion recovery sequence (STIR) magnetic resonance images prior to treatment show the extent of the macrocystic LM with predominant component on the right neck as well as smaller component on the left neck both sparing deeper laryngeal areas (arrow) **c** Periprocedural digital subtraction image during first BEST session, note the puncture of the right neck and filling of macrocystic structures after contrast administration. Partial cyst evacuation to reduce drug dilution and Bleomycin application was performed prior to intralesional electrode placement (arrow). **d** Clinical photographs 1 month after first BEST session, note the significantly reduced swelling, healing superficial components as well as postprocedural skin discolouration (asterisk). **e** Coronal STIR magnetic resonance images 6 months after first BEST session revealing complete regression of the right-sided lesion treated as well as persisting LM components left-sided not yet treated (arrow). **f**, **g** Coronal STIR magnetic resonance images 6 months after second BEST session revealing near-complete regression of all treated LM components (arrows). **h** Clinical photograph 6 months after second BEST session, note completely resolved swelling and residual/partially faded postprocedural skin discolourations

Twenty patients, 10 females and 10 males, with LMs (14/20, 70%) or ld-LVMs (6/20, 30%) treated by BEST were included. Median age at treatment initiation was 12.5 years (range 2–53 years) with 13/20 (65%) paediatric cases (age < 18) that present a median weight of 31 kg (range 12–72 kg) and include four cases with age < 6. The most common LM subtype was microcystic (11/20, 55%), followed by mixed (5/20, 25%), and macrocystic (4/20, 20%), see Fig. 1. Most common indications for treatment were swelling (12/20, 60%), lymphorrhea/bleeding (9/20, 45%), pain (7/20, 35%), superinfection or recurrent erysipelas (5/20, 25%) as well as functional impairment like dysphagia, dyspnoea, or restricted mobility (5/20, 25%). Most involved anatomical areas were head/neck (9/20, 45%) and lower extremities (5/20, 25%), see Table 1. Both therapy-naïve (6/20, 30%) and patients with prior invasive treatment (14/20, 70%) without sufficient symptom improvement were included, either after sclerotherapy (4/20, 20%), surgical debulking (3/20, 15%), or both (6/20, 30%).

**Table 1:** Patient characteristics of study Cohort

Characteristic	Cohort (total, *n* = 20)
	median (range)
Age at treatment	12.5 (2–53)
Female	10/20 (50.0%)
Subtypes of LMs	20/20 (100%)
Microcystic	11/20 (55.0%)
Mixed	5/20 (25.0%)
Macrocystic	4/20 (20.0%)
Involved anatomical areas	
Head and neck	9/20 (45.0%)
Lower extremity	5/20 (25.0%)
Thorax	3/20 (15.0%)
Pelvis	3/20 (15.0%)
Upper extremity	1/20 (5.0%)
Abdomen	1/20 (5.0%)
Treatment rationales	
Swelling	12/20 (60.0%)
Bleeding/Lymphorrhea	9/20 (45.0%)
Pain	7/20 (35.0%)
Superinfection/Erysipela	5/20 (25.0%)
Functional impairment	5/20 (25.0%)
Cosmetic disfigurement	1/20 (5.0%)
Previous invasive treatments	14/20 (70.0%)
Sclerotherapy and surgery	6/20 (30.0%)
Sclerotherapy only	4/20 (20.0%)
Debulking surgery only	3/20 (15.0%)
Others	1/20 (5.0%)

*LMs* lymphatic malformations

### Procedural Characteristics

Patient cohort underwent a total of 27 cycles of BEST with a mean of 1.35 ± 0.6 procedures per patient, as shown Table 2. Seventeen (17/27, 63%) procedures were performed in children. Median bleomycin dose per procedure was 7 mg (range 2–15 mg) with a median of 26 (range 6–110) reversible electroporation cycles. Overall median bleomycin dosage per patient was 7.5 mg (range 2–29 mg). Most common type of electrode used was 15 mm finger (8/27, 29.6%) and 30 mm hexagonal (8/27, 29.6%), see Table 2.

**Table 2:** Procedural data of BEST study

Characteristic	Cohort (total, *n* = 20)	BESTs (total, *n* = 27)
Total BESTs		
1	14/20 (70.0%)	
2	5/20 (25.0%)	
3	1/20 (5.0%)	
Electrode type		
Finger 15 mm		8/27 (29.6%)
Hexagonal 30 mm		8/27 (29.6%)
Finger 20 mm		5/27 (18.5%)
Hexagonal 40 mm		5/27 (18.5%)
Finger 10 mm		3/27 (11.1%)
		median (range)
Dose [mg] of Bleomycin		7 (2–15)
Electroporation cycles^a^		26 (6–110)

*BEST* Bleomycin-electrosclerotherapy.

^a^‘Electroporation cycles’ refer to the number of electrode repositionings

### Patients’ Outcome (Patient-reported questionnaire)

**Fig. 2 A:**
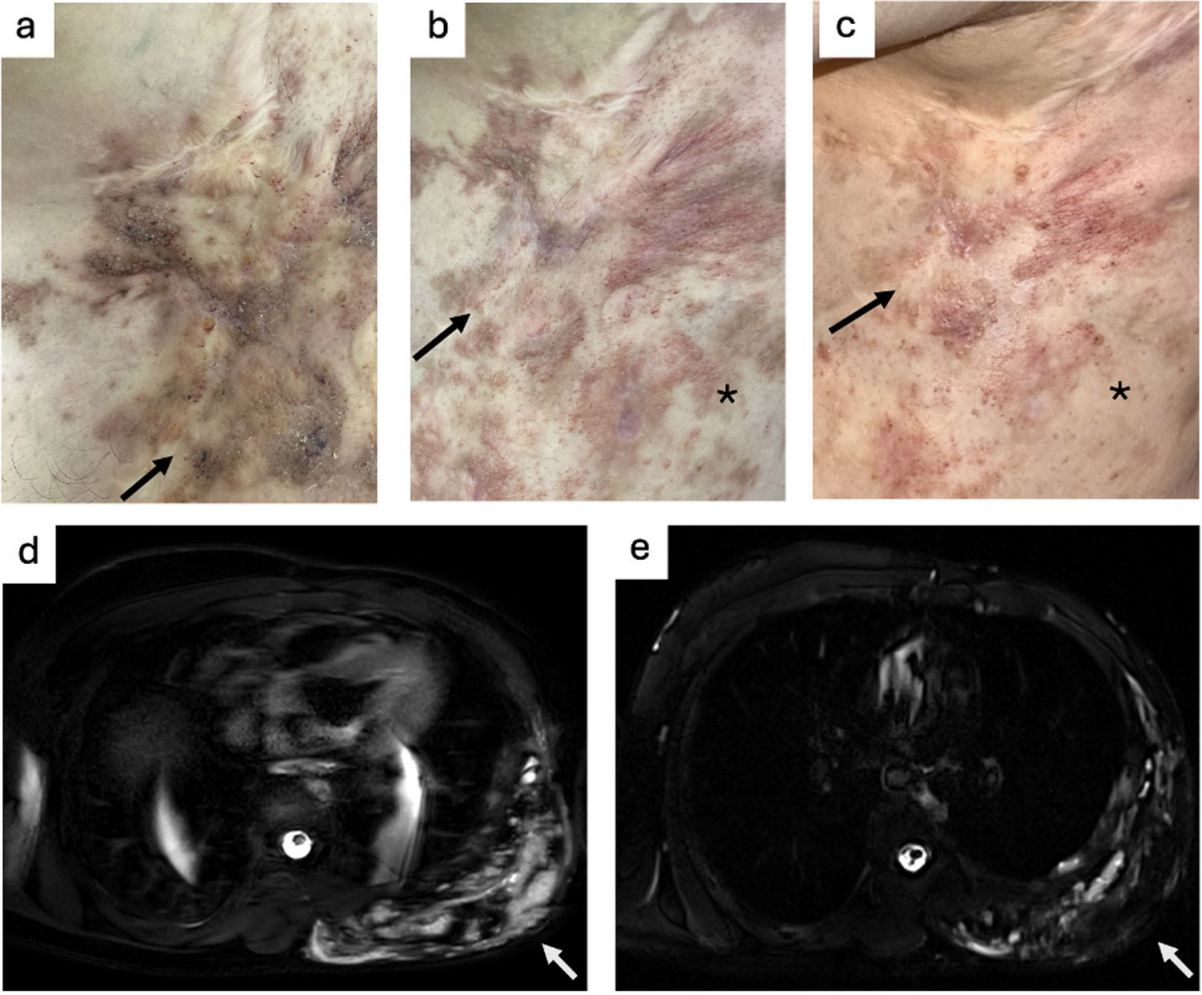
23-year-old male patient with microcystic lymphatic malformations (LMs) of the left treated by two sessions of Bleomycin-electrosclerotherapy (BEST) **a** Clinical photograph before BEST treatment; note the extensive skin involvement including numerous small, translucent and red–purple vesicles, hyperkeratosis with verrucous texture due to chronic lymphatic changes and the mix of red and brownish discolouration following haemosiderin deposition (arrow). Extensive scarring following prior surgical treatment. **b** Clinical photograph 8 weeks after the first BEST session presenting already regressing chronic skin changes (arrow) alongside prominent postprocedural skin discolourations at electrode insertion sites (asterisk). **c** Significantly improved LM-associated chronic skin changes after second BEST session (arrow) as well as almost complete fading of postprocedural skin discolouration (asterisk). **d** T2-weighted magnetic resonance imaging (MRI) sequence showing the extensive microcystic LM of the left trunk before treatment (arrow). e) Follow-up MRI 6 months after second BEST session with significantly regressed findings (arrow), corresponding to the clinical improvement

The mean last follow-up was performed 14.3 ± 9.3 months postprocedural. Complete response was rated by 7/20 (35%) with partial response by 10/20 (50%) and no response by 3/20 (15%) patients. No progression was stated in the postprocedural interval. Sub-analysis revealed no significant difference between clinical response of therapy-naïve and prior invasively treated patients (*p* = 0.56). Changes in subjective health-related QoL were reported as optimal by 10/17 (58.8%), as improved by 4/17 (23.5%), as unchanged by 2/17 (11.8%), and as worsening by 1/17 (5.9%) patients. Three (3/20, 15.0%) patients did not respond to this question. Regarding all patients presented with pain ≥ 1 on the NRS before treatment (13/20, 65%), significant reduction was observed from a median of 6 (3–10) to 2 (0–6) at follow-up. No patient described increased pain severity following BEST. Regarding clinical response, health-related QoL, and pain scales, no significant differences between micro, macrocystic, and mixed subtypes were assessed (*p* = 0.38; *p* = 0.19; *p* = 0.84; *p* = 0.78), as shown Table 3. Postprocedural skin discolourations were reported by 11/20 (55%) patients. While one (1/11, 9.1%) patient-reported unchanged skin discolouration, 7/11 (63.6%) reported reduced, and 3/11 (27.3%) patients stated fully resolved skin discolouration in the postprocedural course (Fig. 2).

**Table 3:** Outcome of Study Cohort

Characteristic	Total cohort (*n* = 20/*n* = 16)	Microcystic (*n* = 11)	Mixed (*n* = 5)	Macrocystic (*n* = 4)	*p*-value
Subjective outcome^a^					0.37^c^
Progression	0/20 (0.0%)	0/11 (0.0%)	0/5 (0.0%)	0/4 (0.0%)	
No response	3/20 (15.0%)	1/11(9.1%)	0 (0.0%)	2/4 (50.0%)	
Partial response	10/20 (50.0%)	5/11 (45.5%)	4 (80.0%)	1/4 (25.0%)	
Complete response	7/20 (35.0%)	5/11 (45.5%)	1 (20.0%)	1/4 (25.0%)	
Health-related QoL^a,b^					0.19^c^
Worsening	1/17 (5.9%)	1/11 (9.1%)	0/5 (0.0%)	0/4 (0.0%)	
Unchanged	2/17 (11.8%)	0/11 (0.0%)	1/5 (20.0%)	1/4 (25.0%)	
Improved	4/17 (23.5%)	1/11 (9.1%)	2/5 (40.0%)	1/4 (25.0%)	
Optimal	10/17 (58.8%)	8/11 (72.7%)	1/5 (20.0%)	1/4 (25.0%)	
Pain VAS before BEST, median (range)^a^	6.0 (3–10)	4 (0–10)	4 (0–8)	4.5 (0–8)	0.84^d^
Pain VAS after, BEST median (range)^a^	2.0 (0–8)	0 (0–6)	2 (0–6)	2 (0–5)	0.78^d^

*BEST* Bleomycin-electrosclerotherapy, *QoL* Quality of life, *VAS* Visual analogue scale

^a^Patient-rated questionnaire (by parents and/or child itself)

^b^Three patients did not respond to this question

^c^Kruskal-Wallis test

^d^Wilcoxon singed rank test

### Imaging Response

Both pre and postprocedural MRI was available in 14/20 (70%) cases. Regarding the remaining 6/20 (30%) cases, all were paediatric patients, in whom only a clinical follow-up was performed to avoid the anaesthesia required for MRI. The mean maximum volume was 793 cm^3^ (IQR 155–2199 cm^3^) before therapy, significantly reduced to 548 cm^3^ (IQR 71–1059 cm^3^) after BEST (*p* = 0.048).

### Safety

Postprocedural complications occurred after 3/27 (11.1%, CIRSE grade 3–4) treatments. After 2/27 (7,4%, CIRSE grade 3) treatments, prolonged intubation and the need for tracheotomy were observed due to extensive and persistent swelling with subsequent airway obstruction. Both patients had a complex involvement of tongue and/or deep paralaryngeal structures. After 1/27 (3.7%, CIRSE grade 4) treatment, a mild sensory impairment in the sole of the foot was reported, already steadily subsiding at 6-month follow-up with a minimal residuum. In our cohort, there were no pulmonary complications periprocedural or during follow-up.

In addition, protective measures were implemented in anticipation of postprocedural swelling; these were not classified as complications. After two sessions, patients with LMs involving deep laryngeal or paratracheal structures were admitted to ICU for a planned period of protective invasive ventilation of up to two postprocedural days. Another patient required parenteral nutrition for 3 days due to expected swelling of pharyngeal structures. Both patients had no unexpected or prolonged airway obstruction and could be transferred to normal ward afterwards without any complications.

## Discussion

This study evaluating BEST for treating LMs and ld-LVMs with predominantly microcystic subtypes revealed high overall clinical response and an acceptable complication rate.

At follow-up, > 80% of patients reported complete or partial clinical response as well as improved health-related QoL while median pain score reduced significantly. Consistent with prior literature on BEST in SFVMs, the results of BEST might surpass those of conventional sclerotherapy [16, 19–21], although direct comparative studies are lacking. Notably, in this study most patients required only one or two treatment sessions for symptom control —fewer than with conventional sclerotherapy [5, 20, 22, 23]. Additionally, BEST appears to offer an excellent minimally invasive option for microcystic LMs which typically do not respond as reliably to conventional sclerotherapy as macrocystic LMs do [20–22]. For example, De Maria et al. reported a 31% cure rate in microcystic lesions versus 53% in macrocystic LMs [24]. In this cohort, all but one patient with microcystic LMs showed a positive response to BEST, highlighting its potential for this difficult-to-treat group [24, 25]. The high therapeutic efficacy in microcystic LMs may be explained since, even if the drug is not injected into every single microcyst, it can still distribute between adjacent cysts due to subsequent electroporation and associated reversible increase in cell membrane permeability. In contrast, conventional sclerotherapy may be generally effective only in those tiny cysts that are directly injected with the sclerosing agent.

Three patients of the cohort showed no response, two of whom had macrocystic subtypes. A possible explanation may be an inhomogeneous electrical pulse distribution leading to incomplete lesion coverage and, particularly in macrocystic subtypes, impaired bleomycin distribution due to haemorrhagic or viscous cyst contents.

MRI demonstrated a significant reduction of mean lesion volume, in line with other studies on BEST and conventional sclerotherapy for SFVMs, although primarily focused on VMs [16, 26–29]. These reports also highlight that clinical and imaging responses often diverge. Spence et al. [30] suggested that partial fibrosis or debulking in certain areas of the malformation may be sufficient to achieve symptom relief. This aligns with clinical observations suggesting that subjective improvement, rather than complete radiological resolution, is the most relevant outcome parameter. Consequently, MRI findings should not serve as the sole criterion for assessing treatment success and should not, in the absence of clinical symptoms, be used in isolation to guide additional treatment sessions. Whether more aggressive BEST treatment strategies, such as more electroporation cycles or higher Bleomycin doses, might reduce recurrence remains an open question and warrants further long-term outcome studies. Similarly, the impact of technical variations in bleomycin administration—such as neat versus foam formulations (e.g., mixed with 1 ml albumin or 1% lidocaine)—on clinical outcomes remains unclear.

The most concerning systemic complication associated with bleomycin is pneumonitis, possibly progressing to irreversible fibrosis. So far, clinically relevant lung injury following sclerotherapy of SFVMs is rare and exclusively reported in VMs [31, 32]. The low incidence of pulmonary adverse events may be attributed to significantly lower doses compared to chemotherapy. Slower pharmacokinetic uptake associated with intralesional—compared to intravenous—administration may further contribute to a safer profile [33, 34]. Nonetheless, it remains essential to minimize the dosage as low as reasonably achievable, especially in young patients typically affected by vascular malformations. Opposed to the median dose of 7 mg in this study, most LMs cohorts were treated using higher doses in conventional sclerotherapy [35, 36].

The overall complication rate of 11% was comparable to conventional sclerotherapy [29, 30], despite reports suggesting that BEST may lead to more pronounced and potentially severe complications [16]. In this cohort, one patient presented mild persistent sensory impairment at the foot sole, possibly due to mechanical nerve injury during BEST. Lesions involving pharyngeal and laryngeal structures form a dedicated subgroup with specific risks, especially airway affection. Thus, for lesions involving the tongue, floor of the mouth, trachea, or larynx, appropriate airway management becomes critical during BEST. Compared to conventional sclerotherapy, BEST appears to induce more pronounced postprocedural swelling [23], reflected by two patients in this study requiring prolonged intubation and tracheostomy. Therefore, patients ’ thorough informed consent regarding potential side effects, along with a well-defined airway management strategy, is essential. Thus, the approach to airway protection must be determined on an individual and interdisciplinary basis, including ENT surgeons and anaesthesiologists, given the considerable variability of lesions with regard to the structures involved and their extent. To broadly mitigate these risks, potential strategies include comprehensive pretreatment imaging to more accurately assess lesion depth and vascularity, real-time airway monitoring, and the use of lower energy electroporation protocols specifically adapted for mucosal tissues. Emerging technical innovations—such as catheter-based localized drug delivery or micro-pulsed electroporation—may further enhance control and safety when treating sensitive anatomical regions. In one case of this cohort, the tracheostomy was retained between sessions to facilitate a second treatment without the need for postprocedural intensive care. Future studies should further evaluate the extent of swelling following BEST compared to conventional sclerotherapy using different sclerosants. From a clinical perspective, a combined approach—using BEST for lesion debulking and conventional sclerotherapy for deeper airway-involving components—may be worth considering.

Postprocedural skin discolouration, a well-known side effect of BEST, was observed in over half of cases. Most patients described at least partial fading at last follow-up, consistent with previous SFVMs data [16]. Although currently there are no published data on permanently lasting skin discolourations, patients and parents need to be consented very clearly about this frequent side effect, especially regarding visible and sensitive regions such as head and neck. Prospective longer-term studies are needed to investigate the underlying mechanisms of skin discolouration in BEST and to elucidate natural course and potential risk of persistence of these discolourations.

Limitations include the retrospective design with potential selection bias, as the data reflect only those available from a single specialized centre. The patient-reported questionnaires, while providing valuable insights into subjective outcomes, were not psychometrically validated, as no validated quality of life assessment tools exist specifically for vascular anomalies. Follow-ups by telephone-based interviews may further introduce recall bias. The inclusion of children < 6 years old resulted in lacking postprocedural MRI due to need of anaesthesia and preferably used US. The overall short duration of follow-up due to the relatively new therapy method allowed no assessment of mid- or long-term recurrence rates following BEST. Further research is required to address long-term responses and to perform direct comparisons of BEST and conventional sclerotherapy to identify patients profiting most from respective treatments.

## Conclusion

BEST is safe and effective for treating LMs and ld-LVMs. BEST has the potential to reduce the number of sclerotherapy cycles and, furthermore, to offer an effective therapeutic alternative for the subgroup of microcystic lesions previously considered difficult to treat.

## Supplementary Information

Below is the link to the electronic supplementary material.

Supplementary file1 (DOCX 15 kb)

Supplementary file2 (PDF 55 kb)
