# Supplementary material for: Safety and Clinical Outcome of Bleomycin-Electrosclerotherapy (BEST) Treating Lymphatic Malformations (LMs)
Source: Cardiovasc Intervent Radiol. 2025 Sep 4;48(10):1428–37. doi: 10.1007/s00270-025-04169-6 (PMC12535933; doi:10.1007/s00270-025-04169-6)
Supplement: Supplementary file 1 — Supplementary file1 (DOCX 15 kb) [file 270_2025_4169_MOESM1_ESM.docx]

**Procedural details**

Treatment was performed under general anaesthesia, TOF 0 relaxation, and FiO_2_ <30%. For macrocystic LMs, the lesion was punctured under US-guidance followed by contrast injection under fluoroscopy-guidance, cyst evacuation to reduce drug dilution, and subsequent intralesional Bleomycin injection. For microcystic LMs, Bleomycin was injected interstitially along the lesion under US-guidance. To ensure homogeneous drug distribution in microcystic lesions, administration is performed during gradual needle retraction, complemented by several punctures as needed based on lesion dimensions. Bleomycin was routinely used in its standard liquid form, diluted to a concentration of 1mg/ml in saline. The administered dose was primarily determined by lesion size: for lesions with a longest diameter of less than 1 cm, up to 0.5 mg was used; for diameters of 1–3 cm, 0.5–1 mg; for 3–5 cm, 1–2 mg; and for lesions larger than 5 cm, more than 2 mg. The maximum bleomycin dose per treatment accepted was 0.2 mg per kg bodyweight, with a cumulative dose <1 mg/kg bodyweight. Subsequently, the electrodes were positioned, and reversible electroporation was started one minute after the bleomycin injection. Electrodes (finger electrodes and hexagonal electrodes) were selected based on configuration, localization, and depth of the lesion while the same procedure was repeated at different locations after percutaneous access, always using a fraction of the bleomycin solution if needed. Overlapping electrode placement was avoided to prevent irreversible electroporation and subsequent tissue necrosis. If possible, the whole lesion volume was covered in one session. The maximum bleomycin dose per treatment accepted was 0.2 mg per kg bodyweight, with a cumulative dose <1 mg/kg bodyweight. To apply the reversible electroporation the electroporation system (Cliniporator™ VITAE, IGEA S.p.A., Carpi, Italy) was used, which provides several independently controlled and isolated outputs, each reaching up to 1000 V/cm (maximum current: 50 A) and generates electrical impulses with a duration of 100 μs between the electrodes.
