# Supplementary material for: Safety and Clinical Outcome of Bleomycin-Electrosclerotherapy (BEST) Treating Lymphatic Malformations (LMs)
Source: Cardiovasc Intervent Radiol. 2025 Sep 4;48(10):1428–37. doi: 10.1007/s00270-025-04169-6 (PMC12535933; doi:10.1007/s00270-025-04169-6)
Supplement: Supplementary file 2 — Supplementary file2 (PDF 55 kb) [file 270_2025_4169_MOESM2_ESM.pdf]

Questionnaire Following Treatment with Bleomycin ElectroSclerotherapy (BEST) for Lymphatic Malformations

Name, First Name ___________________ Date______/______/______

1. Have your symptoms, or those of your child, changed after treatment?
   (Symptoms are recurring infections, pain, asthetic disfigurement, functional impairment, swelling, bleeding)

   ☐ complete response (no symptoms after BEST)
   ☐ partial response (improvement of symptoms)
   ☐ no response (unchanged symptoms)
   ☐ progression (worsening of symptoms)

2. Has your quality of life, or that of your child, changed after treatment with BEST?

   ☐ optimal
   ☐ improved
   ☐ unchanged
   ☐ worsening

3. Did you notice any discoloration ('dark spots') of the skin after the therapy?

   ☐ yes ☐ no

   3.1 If yes, has the discoloration changed over time?

   ☐ unchanged
   ☐ reduced
   ☐ fully resolved

4. Please rate your pain, or that of your child, on a scale from 0 (= no pain) to 10 (= worst imaginable pain) before treatment with BEST.

☐ 1 ☐ 2 ☐ 3 ☐ 4 ☐ 5 ☐ 6 ☐ 7 ☐ 8 ☐ 9 ☐ 10

5. Please rate your pain, or that of your child, on a scale from 0 (= no pain) to 10 (= worst imaginable pain) after treatment with BEST.

☐ 1 ☐ 2 ☐ 3 ☐ 4 ☐ 5 ☐ 6 ☐ 7 ☐ 8 ☐ 9 ☐ 10
